# Neglected Elbow Injury With Severe Flexion Deformity With Myositis Ossificans

**DOI:** 10.7759/cureus.81171

**Published:** 2025-03-25

**Authors:** Hari Krishna Akurathi, Varun Ronanki, Rakesh Kumar Korrapati, Mettu Rami Reddy

**Affiliations:** 1 Medicine, NRI Medical College Chinakakani, Guntur, IND; 2 Pathology, NRI Medical College Chinakakani, Guntur, IND; 3 Orthopaedics, NRI Medical College and General Hospital Chinakakani, Guntur, IND

**Keywords:** arthrolysis, excessive massaging, flexion deformity, heterotopic bone, myositis ossificans

## Abstract

Myositis ossificans is a rare entity of extra-skeletal bone formation in various soft tissues and muscles, often triggered by trauma or injury. We report a case of myositis ossificans traumatica in a 45-year-old female who presented with pain and a fixed flexion deformity of the elbow. Imaging studies, including X-ray and magnetic resonance imaging (MRI), revealed a bony mass in the posterior aspect of the right elbow and presence of triceps injury. The patient underwent surgical excision with arthrolysis. To prevent further heterotopic bone formation, excessive massaging or rubbing of the affected area was avoided. The postoperative period was uneventful.

## Introduction

Myositis ossificans (MO) is a benign non-neoplastic disorder characterized by the formation of heterotopic bone in extra-skeletal soft tissues [1]. Myositis ossificans predominantly affects adolescents and young adults, with the highest incidence occurring in the second and third decades of life. Additionally, males are more commonly affected by MO [1,2]. MO is classified into three types: fibrodysplasia ossificans progressiva (FOP), myositis ossificans circumscripta or traumatica (MOT), and myositis ossificans without a history of trauma (non-traumatic or pseudomalignant MO) [2]. It is primarily an autosomal dominant condition and typically affects a single muscle or muscle group. The quadriceps, brachialis, and thigh adductor muscles are among the most affected regions [1,3,4]. Patients with MO typically present with pain and restricted range of motion following trauma or overuse. The likely causes include repetitive minor mechanical injuries, ischemia, and inflammation [2]. 

## Case presentation

**Figure 1:**
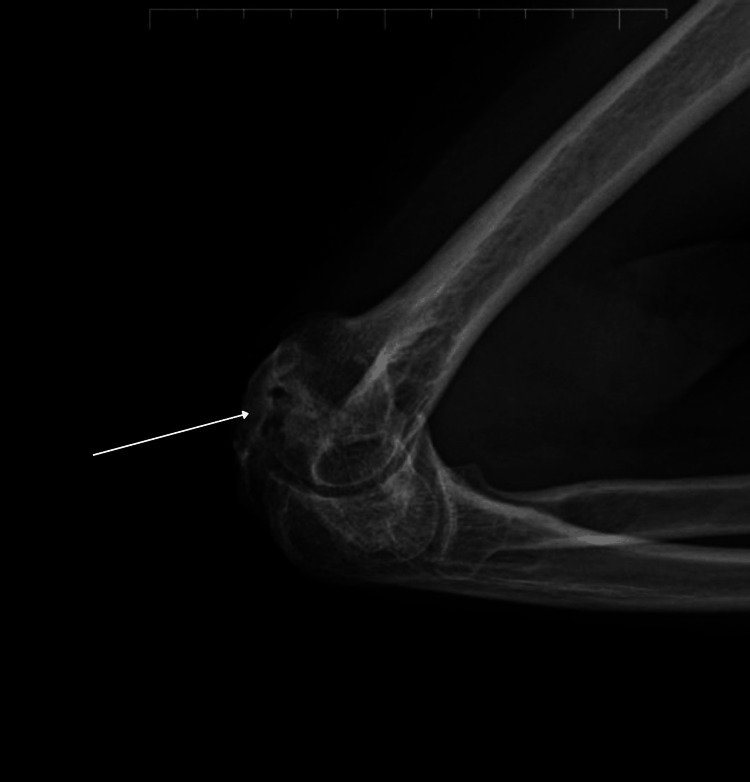
Pre-operative X-rays show ankylosis of elbow joint with bridging bone olecranon and distal humerus antero-posterior (AP) view

**Figure 2:**
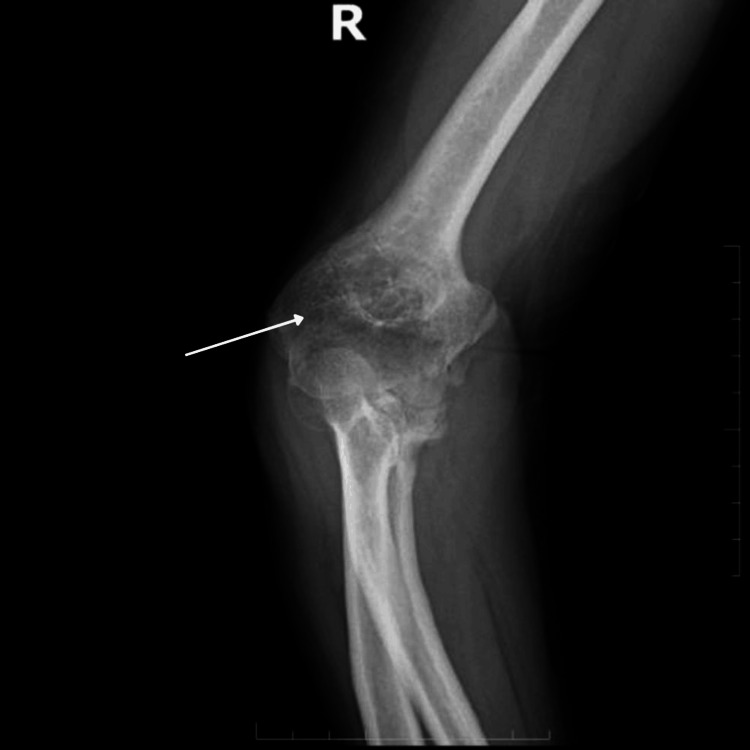
Pre-operative X-rays show ankylosis of elbow joint with bridging bone olecranon and distal humerus lateral view

In this case, a 45-year-old female presented with chief complaint of inability to perform movements with her right elbow for the past two months. She had a history of a fall 16 months prior, during which she sustained no major injuries. However, due to persistent pain, she underwent rigorous physiotherapy eight months ago. Later, movement at the elbow became restricted, with severe flexion deformity. On examination, the right forearm was flexed at the elbow joint, with mild swelling noted over the right olecranon process. Upon palpation, tenderness was elicited over the lateral epicondyle. The neurovascular system of the right upper limb appeared intact. Upon investigations, antero-posterior and lateral X-rays of the right elbow revealed a bony mass extending from the distal humerus to the proximal ulna with bridging of the elbow (humeroulnar) joint as depicted in Figures 1, 2.

**Figure 3:**
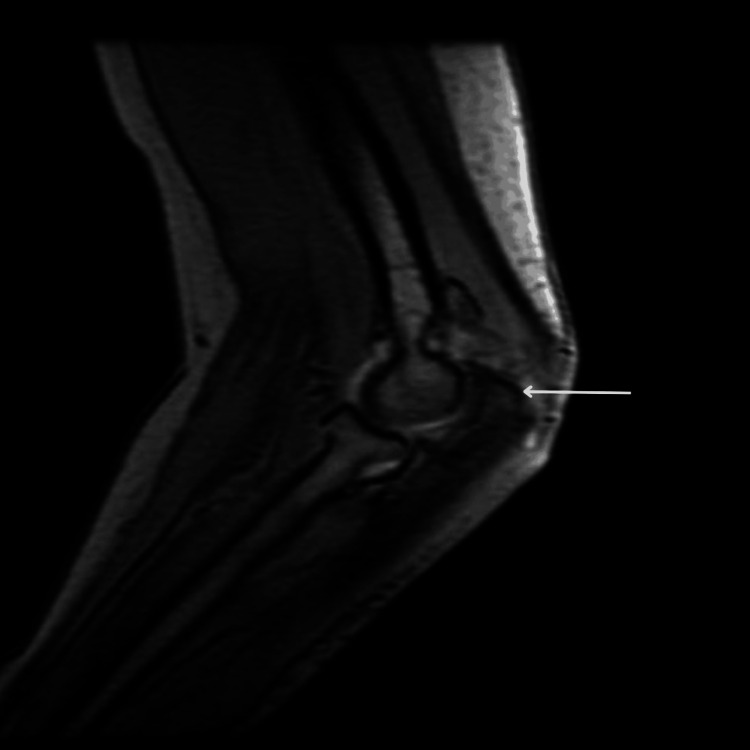
MRI showing heterotopic bone formations

The magnetic resonance imaging (MRI) scan revealed multiple juxta-articular and periarticular ossification areas located in the posterior aspect of the right elbow as in Figure 3.

**Figure 4:**
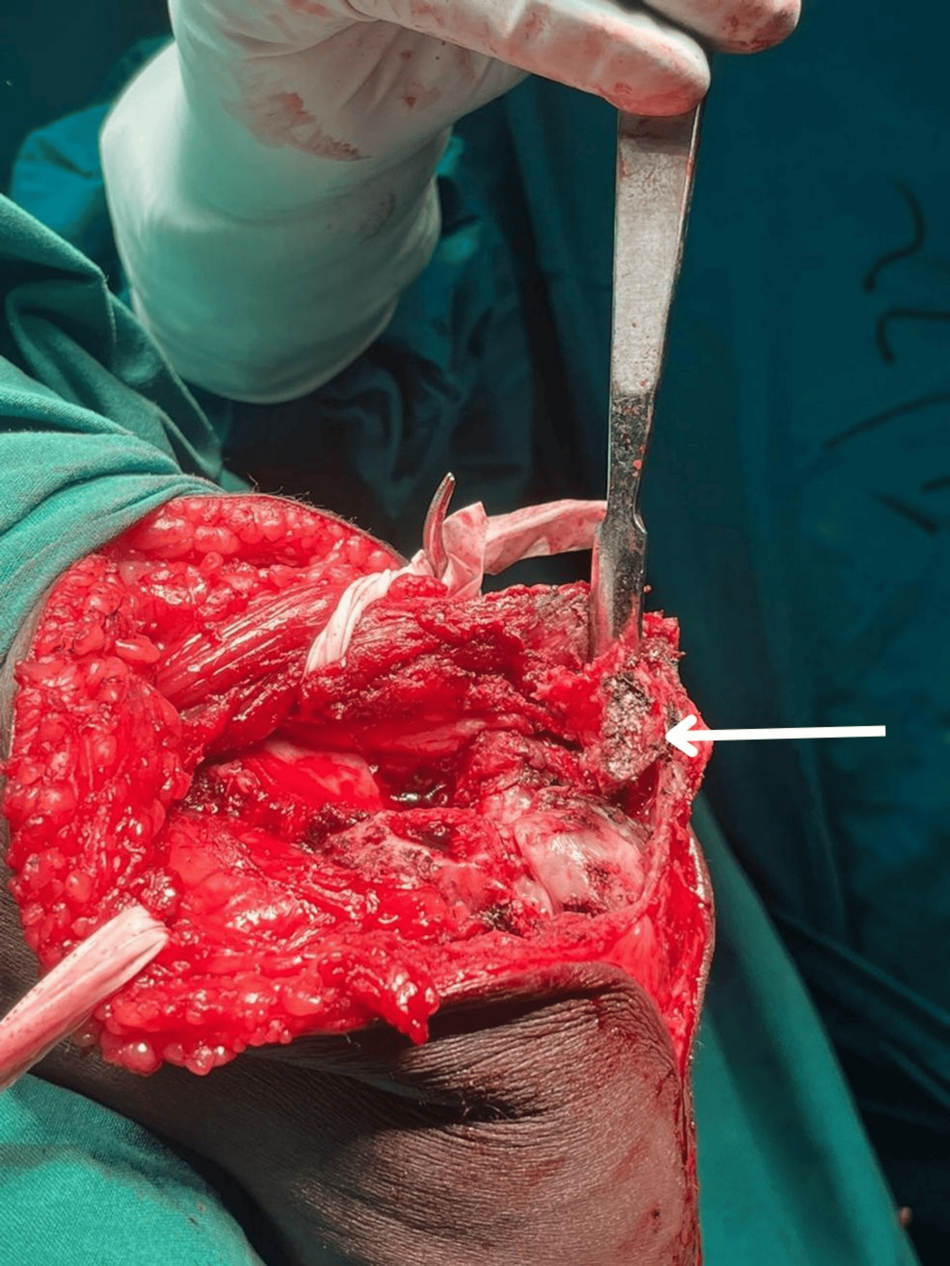
Intra-operative images showing avulsed triceps with heterotopic bone

**Figure 5:**
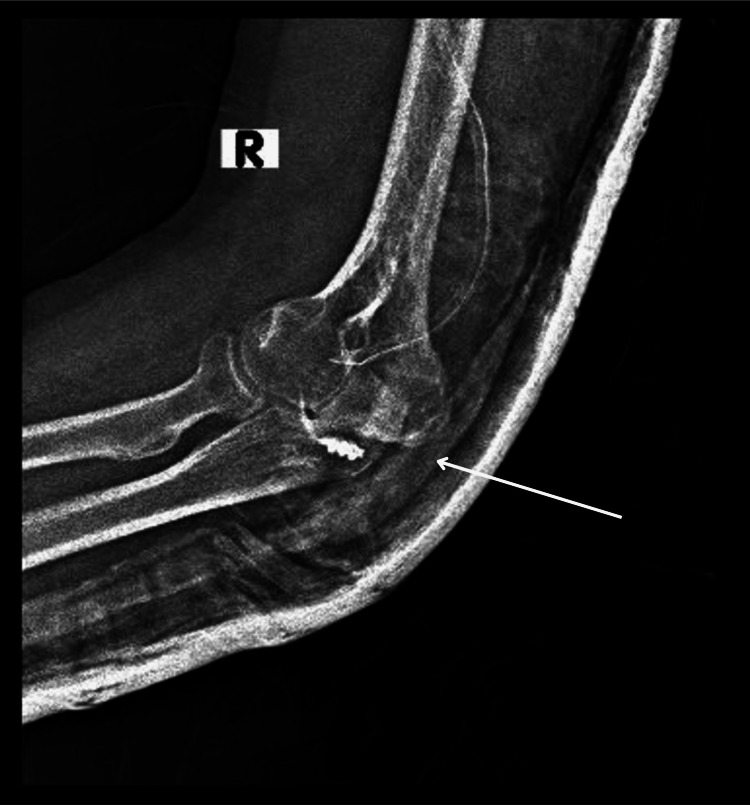
The post-operative X-ray antero-posterior (AP) view showing suture anchor fixation of triceps with heterotopic bone removed

Additionally, osteochondritis dissecans was observed in the capitellum, along with a partial thickness tear of the common flexor origin at the medial epicondyle, along with triceps tear. The complete blood picture (CBP), C-reactive protein (CRP), erythrocyte sedimentation rate (ESR), and other routine investigations were conducted, and all results were within normal limits. Surgical excision with arthrolysis was the treatment of choice. The patient was positioned supine, and a brachial block was administered with the elbow in a lateral position supported by a stand. The surgical site was prepared by scrubbing, draping, and applying a tourniquet. A 12 cm incision was made on the posterior aspect of the elbow, extending towards the lateral aspect. Neurovascular structures were identified and gently retracted, followed by excision of the bone mass. Intraoperative mobilisation of the elbow was done with flexion and extension (Figure 4) and an X-ray was taken after surgery (Figure 5).

**Figure 6:**
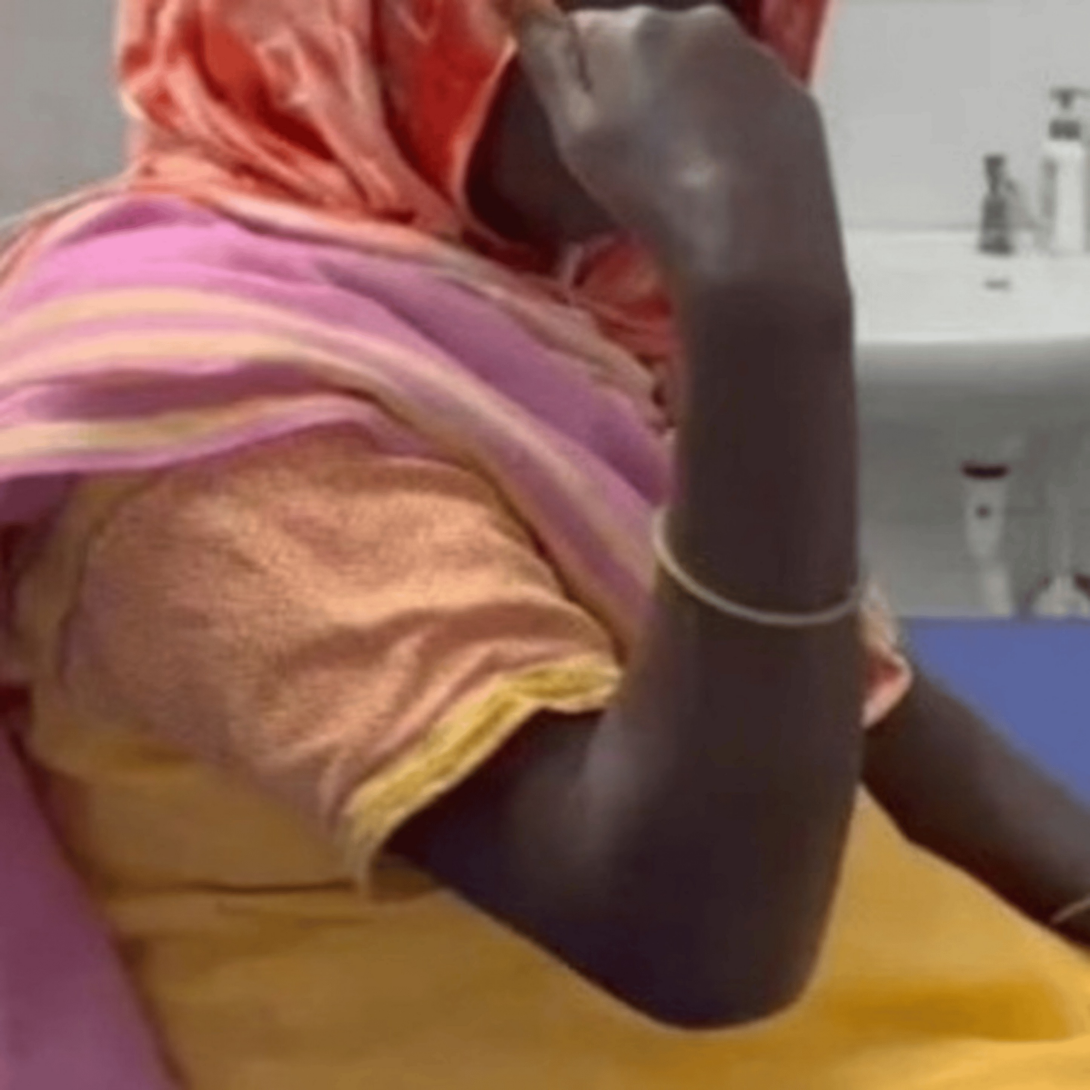
Image showing functional range of motion (ROM) of 65°

The patient is well after surgery and has reported no complaints during follow-up. Follow-up examinations are conducted every four weeks (Figure 6). The patient is still undergoing physiotherapy.

## Discussion

MOT is a condition characterized by the formation of heterotopic bone within muscle or other soft tissues, typically occurring following trauma and hematoma formation in the affected area [3].

Extra-skeletal bone formation was first described by Guy Patin in 1692 and Von Dusche in 1868, named it myositis ossificans traumatica [3].

MOT is primarily caused by changes in perimysial connective tissue rather than the myocytes themselves. Inducible osteoprogenitor cells (IOPCs) in this tissue are responsible for the formation of heterotopic bone in MOT. Under the influence of local and systemic factors, IOPCs have the ability to differentiate into cartilage and bone cells. These cells are not fixed in one location and can migrate through the bloodstream or lymphatic system, being observed in various anatomical sites such as lymph nodes, skin, thymus, and spleen. This ability of IOPCs to form bone in different areas beyond the injury site contributes to the development of MOT [5].

MOT usually occurs in response to traumatic injury without a direct genetic cause, whereas FOP is a rare genetic autosomal dominant disorder caused by mutations on chromosome 2 of the ACVR1 gene. The gene encodes a receptor for a protein called bone morphogenetic protein (BMP), which regulates bone and cartilage formation. This mutation leads to the inappropriate activation of BMP signaling, resulting in abnormal bone formation in soft tissues, often triggered by trauma [6].

In a case series reported by Saad A et al., it was observed that 73% of MO cases affected the muscles of the lower limbs, with 26% affecting the upper limbs. The most affected muscles in the lower limbs were the quadriceps and thigh adductor muscles, while in the upper limbs, the brachialis muscle was frequently involved. Additionally, MO was noted to occur in the pelvis, elbow, and shoulder regions [7].

Typically, MO manifests in young adults who are active in contact sports, with a predominance of males in their second and third decades of life [1,2]. However, in contrast to this typical demographic, our case involves a middle-aged female diagnosed with MO.

MO typically presents with pain, swelling, and a palpable mass at the affected site, accompanied by restricted range of motion and functional impairment of the involved muscle or joint [1].

MO is characterized by the development of distinct zones within the affected tissue, which is often referred to as the "zone phenomena." These zones are considered hallmark features of MO. The outer, middle and inner zones each represent different stages of the process of bone formation within the soft tissue, reflecting the progression of the condition over time. Peripheral or outer zone typically consists of mature lamellar bone with active osteoclasts and a collagenous fibrous stroma. Middle zone contains active osteoblasts and immature osteoid, chondroid, and woven bone tissue. Central or inner zone is characterized by undifferentiated cells, haemorrhagic and necrotic muscular tissue, loose fibrovascular tissue, spindle cells, and prominent giant mesenchymal cells containing abundant mitosis [3].

MOT is diagnosed through a comprehensive approach involving clinical evaluation, imaging studies (such as X-rays and computed tomography (CT) scans), laboratory investigations, and sometimes biopsy.

Radiographic changes can appear as early as two to three weeks post-injury, with definitive bone formation typically visible by two months. Triple-phase bone scans are particularly useful for early detection. CT scans are more sensitive than X-rays for detecting ossification and offer a three-dimensional perspective during the maturation phase of the bone mass. MRI helps localize soft tissue lesions, though its utility diminishes as ossification progresses. Ultrasound (USG) is highly sensitive in detecting the zone phenomenon in early MOT stages before ossifications are visible on other modalities. Biopsy, guided by USG, is crucial for histological confirmation and should encompass the entire lesion. Laboratory tests, including elevated serum alkaline phosphatase levels and erythrocyte sedimentation rate, contribute to the diagnostic workup [8,9].

Treatment strategies for MOT vary based on its stage and severity, including conservative management, physiotherapy, and surgical excision of the heterotopic bone with adjuvant radiotherapy. Conservative approaches include utilizing the RICE principle (Rest, Ice, Compression, Elevation), administering nonsteroidal anti-inflammatory drugs (NSAIDs) such as ibuprofen and naproxen for pain relief, and implementing physiotherapy to maintain or enhance range of motion. However, surgical excision is considered the definitive treatment for symptomatic MOT [7,8]. In our case, surgical excision combined with indomethacin was the chosen treatment approach, however radiotherapy was not done. Post-operative radiotherapy differed due to wound healing problems.

In comparison to the other studies most of the patients are young individuals but in our case along with myositis ossificans there is a triceps tear resulting in variable prognosis despite of surgical excision. Patient may require aggressive physiotherapy for a prolonged period to achieve good results. In our case she was able to do routine activities with mild discomfort.

## Conclusions

Myositis ossificans traumatica is a most debilitating complication of muscle contusion, often leading to severe limitations in joint function. Patients who undergo native treatment and vigorous massage may develop myositis ossificans. It is essential to differentiate MO from other conditions that share similar features, such as malignant bone tumours, soft tissue sarcomas, calcific tendinitis, infectious osteomyelitis, extra-skeletal osteosarcoma, and other types of myositis ossificans. Diagnosing MO requires careful consideration of clinical history, imaging studies (X-ray, CT scan), and sometimes biopsy for histopathological confirmation. Treatment strategies vary depending on the severity and stage of MO, ranging from conservative approaches like rest, NSAIDs, and physical therapy, to surgical excision in refractory cases and adjuvant radiotherapy. Early and accurate diagnosis is crucial to mitigate symptoms, preserve joint function, and optimize outcomes in individuals of MO.

## References

[1] McCarthy EF, Sundaram M (2005). Heterotopic ossification: a review. Skeletal Radiol.

[2] Kougias V, Hatziagorou E, Laliotis N, Kyrvasillis F, Georgopoulou V, Tsanakas J (2019). Two cases of myositis ossificans in children, after prolonged immobilization. J Musculoskelet Neuronal Interact.

[3] Shaik KV, Chari H (2017). Traumatic myositis ossificans: treatment protocol and review of literature. Int J Sci Res.

[4] Orava S, Sinikumpu JJ, Sarimo J, Lempainen L, Mann G, Hetsroni I (2017). Surgical excision of symptomatic mature posttraumatic myositis ossificans: characteristics and outcomes in 32 athletes. Knee Surg Sports Traumatol Arthrosc.

[5] Nägele M, Hamann M, Koch WF (1996). Myositis ossificans. Muscle Imaging in Health and Disease.

[6] Talbi S, Aradoini N, Mezouar IE, Abourazzak FE, Harzy T (2016). Myositis ossificans progressive: case report. Pan Afr Med J.

[7] Saad A, Azzopardi C, Patel A, Davies AM, Botchu R (2021). Myositis ossificans revisited - the largest reported case series. J Clin Orthop Trauma.

[8] Sodl JF, Bassora R, Huffman GR, Keenan MA (2008). Traumatic myositis ossificans as a result of college fraternity hazing. Clin Orthop Relat Res.

[9] Lacout A, Jarraya M, Marcy PY, Thariat J, Carlier RY (2012). Myositis ossificans imaging: keys to successful diagnosis. Indian J Radiol Imaging.

